# A New Strategy for Glioblastoma Treatment: In Vitro and In Vivo Preclinical Characterization of Si306, a Pyrazolo[3,4-*d*]Pyrimidine Dual Src/P-Glycoprotein Inhibitor

**DOI:** 10.3390/cancers11060848

**Published:** 2019-06-19

**Authors:** Anna Lucia Fallacara, Claudio Zamperini, Ana Podolski-Renić, Jelena Dinić, Tijana Stanković, Marija Stepanović, Arianna Mancini, Enrico Rango, Giulia Iovenitti, Alessio Molinari, Francesca Bugli, Maurizio Sanguinetti, Riccardo Torelli, Maurizio Martini, Laura Maccari, Massimo Valoti, Elena Dreassi, Maurizio Botta, Milica Pešić, Silvia Schenone

**Affiliations:** 1 Dipartimento Biotecnologie, Chimica e Farmacia, Università degli Studi di Siena, 53100 Siena, Italy; al.fallacara@gmail.com (A.L.F.); claudiozamperini@yahoo.it (C.Z.); arianna.mancini3@gmail.com (A.M.); rango.enrico@gmail.com (E.R.); giulia.iovenitti@unisi.it (G.I.); molinari.a2@gmail.com (A.M.); 2 Lead Discovery Siena S.r.l., via Vittorio Alfieri 31, Castelnuovo Berardenga, 53019 Siena, Italy; l.maccari@leaddiscoverysiena.it; 3 Department of Neurobiology, Institute for Biological Research “Siniša Stanković” (IBISS), University of Belgrade, 11060 Belgrade (RS), Serbia; ana.podolski@ibiss.bg.ac.rs (A.P.-R.); jelena.dinic@ibiss.bg.ac.rs (J.D.); tijana.andjelkovic@ibiss.bg.ac.rs (T.S.); marija.stepanovic@ibiss.bg.ac.rs (M.S.); camala@ibiss.bg.ac.rs (M.P.); 4 Department of Pharmacy, University of Pisa, 56126 Pisa, Italy; 5 Dipartimento di Scienze di Laboratorio e Infettivologiche, Fondazione Policlinico Universitario “A. Gemelli” IRCCS, 00168 Rome, Italy; francesca.bugli@unicatt.it (F.B.); maurizio.sanguinetti@unicatt.it (M.S.); torelliric@yahoo.it (R.T.); maurizio.martini@unicatt.it (M.M.); 6 Istituto di Microbiologia, Università Cattolica del Sacro Cuore, 00168 Rome, Italy; 7 Dipartimento Scienze della Vita, Università degli Studi di Siena, 53100 Siena, Italy; massimo.valoti@unisi.it; 8 Department of Pharmacy, Università degli Studi di Genova, 16132 Genova, Italy; schenone@difar.unige.it

**Keywords:** glioblastoma, multidrug resistance, P-gp inhibitors, Src inhibitors, in vitro ADME, pharmacokinetics, brain distribution, tolerability

## Abstract

Overexpression of P-glycoprotein (P-gp) and other ATP-binding cassette (ABC) transporters in multidrug resistant (MDR) cancer cells is responsible for the reduction of intracellular drug accumulation, thus decreasing the efficacy of chemotherapeutics. P-gp is also found at endothelial cells’ membrane of the blood-brain barrier, where it limits drug delivery to central nervous system (CNS) tumors. We have previously developed a set of pyrazolo[3,4-*d*]pyrimidines and their prodrugs as novel Src tyrosine kinase inhibitors (TKIs), showing a significant activity against CNS tumors in in vivo. Here we investigated the interaction of the most promising pair of drug/prodrug with P-gp at the cellular level. The tested compounds were found to increase the intracellular accumulation of Rho 123, and to enhance the efficacy of paclitaxel in P-gp overexpressing cells. Encouraging pharmacokinetics properties and tolerability in vivo were also observed. Our findings revealed a novel role of pyrazolo[3,4-*d*]pyrimidines which may be useful for developing a new effective therapy in MDR cancer treatment, particularly against glioblastoma.

## 1. Introduction

Multidrug resistance (MDR) is a severe and pervasive clinical problem which actually represents the leading cause of cancer treatment failure. MDR occurs when cancer cells develop resistance to structurally and mechanistically unrelated classes of anticancer drugs [1] and involves several complex mechanisms [2], among which the overexpression of ATP-binding cassette (ABC) transporters is the most prominent. ABC transporters regulate the ATP-dependent efflux of toxic endogenous molecules and chemotherapeutics out of the cell. Among the 49 ABC transporters known so far, P-glycoprotein (P-gp, MDR1, ABCB1), multi-drug resistance protein 1 (MRP1, ABCC1) and breast cancer resistance protein (BCRP, ABCG2) appear to play the most important roles in inducing resistance in several cancers, such as lung, breast, colon, ovarian, central nervous system (CNS) cancers, and melanomas [3,4,5]. Moreover, ABC efflux transporters are also found in excretory epithelial cells of the gastrointestinal tract (GI), kidney, liver, and in endothelial cells of physiological barriers such as the blood-brain barrier (BBB) [6,7]. Thus, drug efflux pumps are key elements for drug delivery and efficacy in target tumor cells, as well as for CNS distribution. Therefore, their modulation offers a novel strategy to enhance the penetration and improve the efficacy of drugs into the brain, especially for drug-resistant CNS tumors [8].

Glioblastoma Multiforme (GBM) is the most aggressive (World Health Organization grade IV) brain tumor in adults and the most frequent one. The majority of GBM patients do not live for longer than 1 year [9] and the average 5-year survival rate for patients is less than 3% [10]. After standard treatment, which requires surgical resection followed by concomitant radiotherapy and temozolomide therapy, fatal relapses commonly occur, and recurrent GBM tends to be even more aggressive and resistant to medical treatment than its primary counterpart [11].

In the past two decades, molecular characterization of genomic alterations in GBM has offered an array of altered pathways that can be specifically targeted. Given their pivotal role in tumorigenesis, tyrosine kinases (TKs) have been indicated as an interesting target to treat several cancers [12]. Abnormal or deregulated activity of at least one TK is responsible for oncogenic activity across a broad range of tumors, and in particular of 67.3% of GBM [13]. Coordinately, small-molecule compounds that inhibit the kinase domain of specific TKs have been widely introduced into clinical practice for a number of advanced solid cancers in the last two decades [14]. However, to date, kinase inhibitors have shown minimal efficacy in GBM clinical trials due to the unique characteristics of GBM: (i.) its infiltrative, invasive, and aggressive nature; (ii.) intrinsic and/or acquired drug resistance; (iii.) unsuccessful drug delivery across BBB [15].

In the last years, we have focused on the development of novel TK inhibitors based on the pyrazolo[3,4-*d*]pyrimidine scaffold. These compounds act as ATP-competitive tyrosine kinase inhibitors [16] against the Src-Family Kinases (SFKs) [17]. SFKs are involved in the regulation of cell proliferation, survival, invasion and angiogenesis, thus playing a crucial role in the development and progression of many cancer types, including GBM [18]. In particular, elevated Src activity/phosphorylation are found in GBM samples and cancer cell lines [19,20].

The implication of SFKs in glioma biology has led to the clinical evaluation of the SFK inhibitor- dasatinib, as a novel therapeutic option in particular for recurrent GBM [21,22,23]. However, its usefulness was limited by pharmacokinetics aspects, i.e., short half-life, sub-optimal CNS penetration, and inadequate delivery to the tumor. In vitro and in vivo studies have demonstrated that dasatinib is a substrate for the efflux transporters P-gp and BCRP, which are highly expressed in the BBB [24,25] and GBM cells [26].

It was shown that TKIs interact with efflux transporters either as their substrates or inhibitors. In some cases, TKIs which are substrates for efflux transporters, when applied in high concentrations, can act as inhibitors of transporters [27,28,29,30]. Some of TKIs acting as ABC transporters inhibitors either entered clinical trials or were clinically approved [31,32,33]. TKIs may also modulate MDR by regulating the expression levels of MDR proteins [34]. Considering the dual inhibitory potential of TKIs, these anticancer agents are valuable for combining with other chemotherapeutics [35].

The inhibition of drug efflux transporters often alters the pharmacokinetic properties of the co-administered substrate drug. The increase of oral bioavailability or decrease in biliary excretion and clearance usually result in higher systemic exposure and toxicity. Thus, lapatinib and gefitinib have been reported to strengthen the bioavailability of certain antitumor agents [36,37].

**Figure 1:**
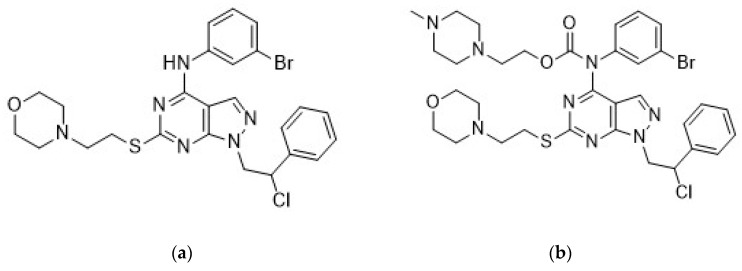
Molecular structure of Si306 (**a**) and pro-Si306 (**b**).

The anticancer activity of pyrazolo[3,4-*d*]pyrimidines has been proved in vitro against several cancer cell lines, both from solid tumors (osteosarcoma [38], prostate [39], neuroblastoma [40,41], glioblastoma [42,43], rhabdomyosarcoma [44], mesothelioma [45], medulloblastoma [46], medullary thyroid carcinoma [47]) and hematological tumors (leukemia [48] and Burkitt lymphomas [49]), and further confirmed by in vivo studies in mouse models of neuroblastoma [40], leukemia [48], and glioblastoma [50]. In particular, the combination of compound Si306 (Figure 1a), a pyrazolo[3,4-*d*]pyrimidine derivative with radiotherapy strongly potentiated the suppression of U87 xenograft growth in nude mice with respect to control and single treatments [50]. Moreover, following intraperitoneal (i.p.) injection, Si306 brain concentration progressively increased during the following 24 h, and the compound appeared to accumulate in the brain. Single oral treatment with Si306 prolonged by 30% the survival of mice orthotopically injected with U87 cells [50]. Overall, this evidence confirmed the ability of Si306 to efficiently reach the brain. Analogous results were obtained with the prodrug of Si306 (pro-Si306, Figure 1b), which showed an enhanced solubility and good efficacy in an orthotopic model of GBM with respect to the parental drug [50].

In this study, we investigated the effect of Si306 and its prodrug on P-gp-mediated efflux, interaction with cytochrome P450 isoform 3A4 (CYP3A4), and suggested their potential use as sensitizers in combination with chemotherapeutics. Additionally, in order to widen the characterization of Si306, which may be useful for the design of preclinical studies, we explored its plasma and brain pharmacokinetics after both oral (p.o.) administration and intravenous (i.v.) injection, including plasma protein binding, and its acute toxicity in mice.

## 2. Results

### 2.1. Cell Growth Inhibition by Dasatinib, Si306 and Pro-Si306

The effects of c-Src inhibitors on GBM cell growth were evaluated by the MTT assay. The results obtained in sensitive LN-229, -U87 and MDR -U87-TxR cell lines after 72 h treatment are shown in Table 1.

Si306 and pro-Si306 exerted identical growth inhibitory effect in U87 cells with approximate IC_50_ values of 3 µM. Pro-Si306 retained unchanged efficacy in U87-TxR cells and LN-229, while IC_50_ for Si306 increased to 4.8 µM for U87-TxR and to 8.0 µM for LN-229. The IC_50_ values for dasatinib were 6.1 µM, 8.5 µM and 24.7 µM in U87, U87-TxR and LN-229 cells, respectively. Cell viability inhibition by Si306 and pro-Si306 was evaluated also on other GBM cell lines and results are shown in Table S1.

### 2.2. Influence of c-Src Inhibitors on P-gp Expression and Activity

**Figure 2:**
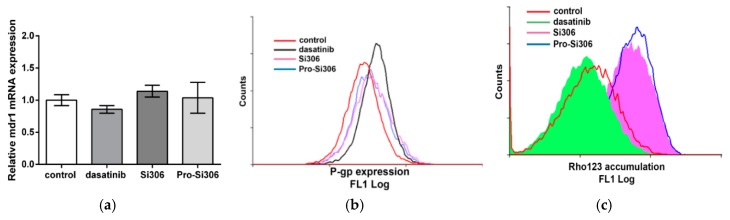
The effects of c-Src inhibitors on P-gp expression and function. (**a**) qRT-PCR analysis of mRNA mdr1 expression (mean ± S.D., *n* = 3) and (**b**) flow-cytometric profiles of P-gp expression in U87-TxR cells after 72 h treatment with 5 µM Si306, pro-Si306 and dasatinib. (**c**) Flow-cytometric profile of Rho 123 accumulation after 30 min treatment with 5 µM Si306, pro-Si306 and dasatinib in U87-TxR cells.

The expression of mRNA mdr1 was unaffected by all examined c-Src inhibitors (Figure 2a), while only dasatinib increased P-gp expression (Figure 2b). According to rhodamine (Rho) 123 (fluorescent P-gp substrate) intracellular accumulation after 30 min, Si306 and its prodrug considerably inhibited P-gp activity, while dasatinib did not show a similar activity (Figure 2c).

**Figure 3:**
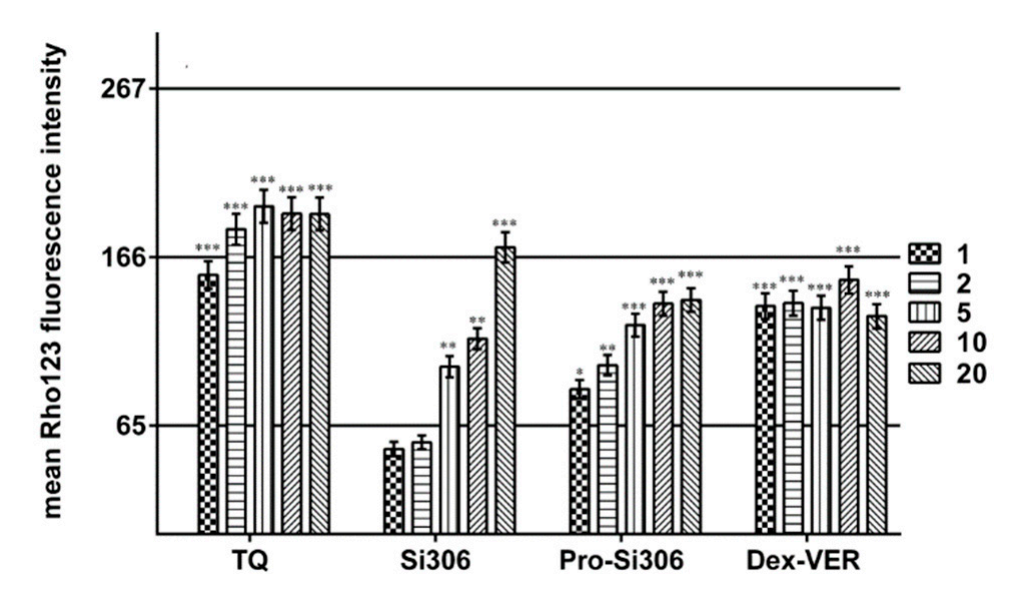
Dose-dependent inhibition of P-gp function. Rho 123 accumulation in U87-TxR cells (values expressed as mean fluorescence intensity ± S.D.) was assessed after application of different concentrations of Si306, pro-Si306, Dex-VER (1, 2, 5, 10, 20 µM) and TQ (1, 2, 5, 10, 20 nM). The mean Rho 123 accumulation of sensitive cells—U87 was set as the upper limit (166), while the mean Rho 123 accumulation of MDR cells—U87-TxR was set as the lower limit (65). IC_50_ values were calculated between these two limits. Two independent experiments were performed (a minimum of 10,000 events was collected for each experimental sample). The significant difference to the lower limit (Rho 123 accumulation in U87-TxR cells) is expressed as: **, *p* < 0.01; ***, *p* < 0.001.

Next, we determined the concentration of c-Src inhibitors necessary to inhibit P-gp activity by 50% (IC_50_ of P-gp inhibition, Table 2) We examined the accumulation of Rho 123 in sensitive -U87 and MDR-U87-TxR GBM cells after 30 min and compared this accumulation with that obtained after treatment with increasing concentrations of Si306 and pro-Si306 (Figure 3). The effects were compared with those promoted by the well-known P-gp inhibitors tariquidar (TQ) and Dex-verapamil (Dex-VER) at the same TK inhibitor concentrations. Si306 and pro-Si306 led to the inhibition of P-gp function in a concentration-dependent manner illustrated by the progressive increase in Rho 123 accumulation (Figure 3).

The methodology used for the calculation of IC_50_ values for P-gp inhibition is described in detail in Figure S1.

The IC_50_ values of P-gp inhibition for Si306 and pro-Si306 were 8.5 µM and 3.8 µM, respectively (Table 2). TQ and Dex-VER did not display a dose-dependent effect as their first applied concentration surpassed the calculated IC_50_ values of 0.3 nM and 0.7 µM, respectively (Figure 3 and Table 2).

### 2.3. Reversion of Paclitaxel Resistance by c-Src Inhibitors

**Figure 4:**
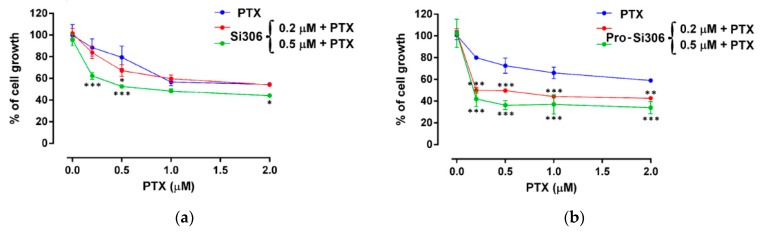
c-Src inhibitors enhance the sensitivity of U87-TxR cells to paclitaxel (PTX). The effects of simultaneous combinations of Si306 (**a**) and pro-Si306 (**b**) with PTX on U87-TxR cell growth inhibition were assessed by 3-(4,5-dimethylthiazol-2-yl)-2,5-diphenyltetrazolium bromide (MTT) assay; values are expressed as mean ± S.D. (*n* = 3). The significant difference to untreated control is shown as: *, *p* < 0.05; **, *p* < 0.01; ***, *p* < 0.001.

Considering the potential of Si306 and pro-Si306 to inhibit P-gp function, we examined their ability to reverse paclitaxel (PTX) resistance in U87-TxR cells (Figure 4 and Table 3).

The effects of the simultaneous combination of our compounds with PTX were assessed by the MTT assay. Both c-Src inhibitors applied at low concentrations (0.2 µM and 0.5 µM) enhanced the PTX efficacy, as indicated by a significant increase in the relative reversal index (Table 3).

### 2.4. Si306 and pro-Si306 Interaction with CYP3A4 in Human Liver Microsomes

**Figure 5:**
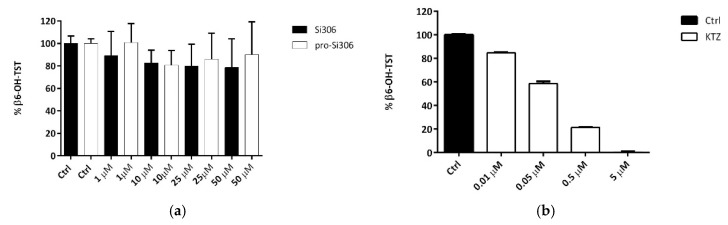
Inhibition of CYP3A4 isoform activity by Si306 and pro-Si306 (**a**) and ketoconazole (KTZ, **b**), measured as % β6-hydroxytestosterone (β6-OH-TST) mean ± S.D., compared to the control (Ctrl) mix in absence of compounds (*n* = 3).

Si306 and pro-Si306 interaction with CYP3A4 isoform has been evaluated in vitro using testosterone as probe through the quantification of its CYP-3A4 metabolite 6β-hydroxytestosterone in presence or absence of different concentrations (1–50 μM range) of Si306 and pro-Si306. At the highest concentration used the maximum of inhibition (20%, not significative) was promoted by Si306, while pro-Si306 inhibited the testosterone hydroxylation at a lower concentration (−15%) (Figure 5a). Ketoconazole was used as standard CYP3A4 inhibitor to validate our method [51] (Figure 5b).

### 2.5. Protein-Binding Profile of Si306 and pro-Si306 to HSA and AGP

**Figure 6:**
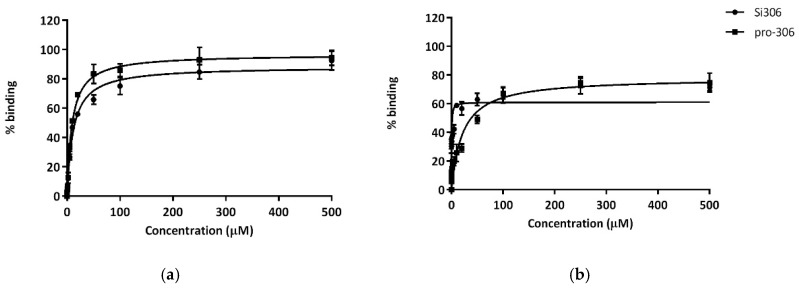
Binding % curves corresponding to the best fit ± S.D. of the data referring to the fluorescence quenching of AGP (**a**) and HSA (**b**) exposed to a 0–500 µM concentration range of Si306 and pro-Si306 which is reported on the x axis (*n* = 3).

The plasmatic protein-binding profiles of Si306 and pro-Si306 in 10 μM Human Serum Albumin (HSA) and Alpha-1-acid glycoprotein (AGP) solutions are illustrated in Figure 6.

Both Si306 and its prodrug presented a one-site binding kinetics for HSA and AGP. As shown, Si306 displayed a high affinity for HSA and AGP (K_D_ 0.35 and 11.49 µM respectively, Table 4). On the other hand, pro-Si306 demonstrated a lower affinity for HSA (K_D_ 21.45 μM, Table 4). However, for AGP, K_D_ values for Si306 and pro-Si306 were comparable (K_D_ 9.28 μM, Table 4).

Our method was validated by using paracetamol (low affinity to plasmatic proteins), diazepam (medium affinity) and warfarin (high affinity) as standard ligands and by referring to results that were previously reported [52].

### 2.6. Pharmacokinetics and Biodistribution of Si306 After i.v. and per os Administration

A pharmacokinetic and biodistribution study was performed in order to further investigate if the observed in vitro inhibitory activity of Si306 against P-gp could significantly affect its gastrointestinal (GI) absorption and its brain penetration. For this purpose, the PK profile of Si306 was studied after i.v. and p.o. administration. Si306 was administrated as a single dose at 25 mg/kg and 50 mg/kg i.v. and p.o. in a final volume of 200 μL and 400 μL of vehicle, respectively. Blood and organs were collected from mice and the concentration of Si306 was determined by HPLC-MS analysis. Table 5 shows the recovery and matrix effect data, which suggest that the developed analysis method could be successfully applied to the pharmacokinetic and biodistribution studies for Si306.

**Figure 7:**
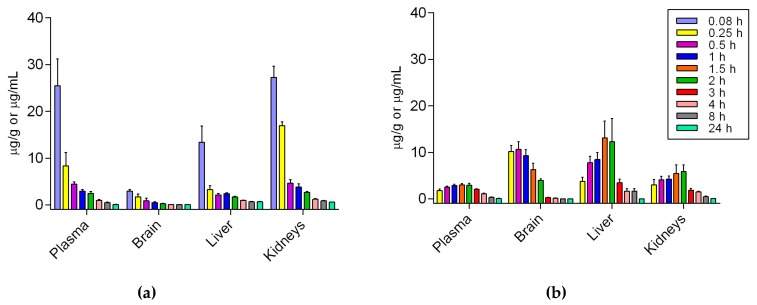
Pharmacokinetics of Si306 (mean ± S.E.M., *n* = 5, plasma: µg/mL; tissues: µg/g). (**a**) Distribution in mice tissues after i.v. administration over 24 h. (**b**) Distribution in mice tissues after p.o. administration over 24 h.

The plasma concentration of Si306 has been reported in Figure 7, while the corresponding PK parameters are shown in Table 6.

Following oral administration, Si306 was rapidly absorbed with a C_max_ of 2.97 μg/mL after 1.5 h (T_max_) while the observed C_max_ for i.v. treatment was 8.5 times higher. However, either AUC and MRT showed similar values after the two different administration (see Table 6). The plasma half-life (t_1/2_) also remains in the same range of values being 5.15 h and 5.96 h after i.v. and p.o. administration, respectively. Moreover, the oral bioavailability of Si306 has been estimated as 33.3% using the following equation (1), confirming a good absorption level from the GI apparatus.

F_p.os_ = [(AUC_p.os_ x D_i.v._)/(AUC_i.v._ x D_p.os_)] × 100 (1)

In both cases, Si306 was able to reach the brain. In particular, after i.v. injection, the maximum concentration into the brain (2.94 µg/g) has been detected immediately after 5 min, indicating a faster penetration of the BBB (Figure 7a). Interestingly, after oral administration a large amount of Si306 has been detected (10.65 µg/g) into the brain after 30 min, then the concentration slowly decreased during the next 24 h (Figure 7b). Moreover, the AUC brain to plasma ratio parameter confirmed the brain distribution to be greater for the oral route (1.185) with respect to i.v. (0.095).

An immediate distribution followed by a fast concentration decrease in a time-dependent manner was also observed for liver and kidneys when Si306 was administered intravenously (Figure 7a). On the other hand, orally administered Si306 showed its C_max_ in liver and kidneys at 1.5 h and 2 h respectively (Figure 7b).

### 2.7. Acute Toxicity Study in Mice

**Figure 8:**
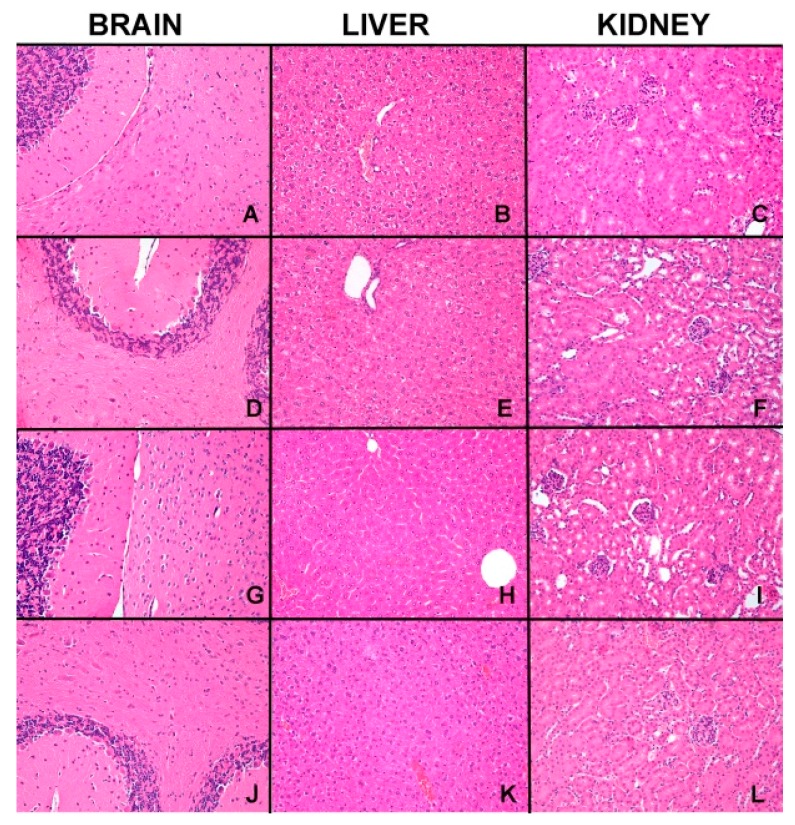
The figure shows representative microscopic pictures (200× magnification) of kidney, liver and central nervous system tissues of untreated mice (**A**–**C**); mice treated with only vehicle (**D**–**F**); mice treated with dose level of 50 mg/kg of Si306 (**G**–**I**) and mice treated with the dose level of 100 mg/kg of Si306 (**J**–**L**).

The acute toxicity study provided useful information concerning the effect of acute exposure of test animals to high doses of the compound under investigation. Treatment of mice with Si306 did not produce treatment-related mortality at the limit test dose (100 mg/kg), and besides, throughout the four days observation period, no significant changes occurred in the behavior, such as apathy, hyperactivity, vomiting, diarrhea, and morbidity, among the tested animals. Thus, Si306 was found to be safe at the dose level of 100 mg/kg and consequently, the LD_50_ value for i.v. toxicity is considered to be more than 100 mg/kg. During the experiment, all animals were examined daily for any clinical signs and no behavioral alteration such as motor activity, posture or autonomic features were observed. The body weights of all mice recorded before the experiment and once every day after the treatment showed no significant changes. No microscopic alterations were found in the main metabolic organs (liver and kidney) and in the target organ of the drug (central nervous system) in treated versus untreated mice (Figure 8).

## 3. Discussion

In the last ten years, pharmaceutical research in oncology has produced many agents targeting important pathways that are considered as being crucial for the development and progression of different tumor types. Although these targeted agents have shown impressive preclinical results, the real clinical benefits have not been as satisfying as expected. It is clear that a great deal of effort was invested into understanding the molecular mechanisms involved in cancer development, while the evaluation of drug interactions with cellular components different from the target molecule was neglected.

New TKIs are being approved from the FDA each year on a regular basis [53], either given alone or in combination with conventional chemotherapeutics. Although TKIs have revolutionized cancer therapy, increasing resistance to TKIs has been documented [54]. Enhanced efflux of TKIs due to the over-expression of P-gp and BCRP in cancer cells is usually responsible for the poor chemotherapeutic response [55,56,57]. Accumulating evidence continues to show that numerous TKIs are substrates of P-gp, so once they are entered into the cells, they are pumped out by ABC transporters [58]. Therefore, the ability of a TKI drug to inhibit P-gp is considered as an important feature for successful anticancer treatment, especially in the case of GBM, whose therapy is additionally compromised by the presence of the BBB.

Recently, we reported an exploratory pharmacokinetic study after i.p. injection of Si306 and pro-Si306 [50]. The data showed a prolonged overall exposure to the active Si306 after the injection of the prodrug, probably due to the slow hydrolysis into the blood of pro-Si306. The concentration of prodrug quantified into the brain was found higher than the quantity of Si306 measured in the brain of mice treated only with the free drug. Moreover, in comparison to the parental drug, after p.o. administration, pro-Si306 demonstrated a comparable efficacy with a slightly increasing median survival time of mice in an orthotopic GBM model [50]. Finally, the PK profile of pro-Si306 administrated p.o. was evaluated. Differently from i.p. injection, pro-Si306 was detected neither into the plasma nor in the brain, probably because of a complete hydrolysis occurred into the GI tract [50].

Considering the results obtained in our previous study, herein we decided to evaluate the effect of Si306 and pro-Si306 on P-gp efflux pump, which accounts for GI absorption and BBB penetration of different substrates, including anticancer drugs and TKIs. The effect of our compounds has been investigated compared with a reference compound, the well-known TKI dasatinib. The cell growth inhibition reported for dasatinib ranges between 7 and 50 μM in GBM cell lines [59]. These concentrations are above those clinically relevant. We showed that the novel c-Src inhibitor Si306 and its prodrug possess higher cell growth inhibitory potential with almost double efficacy in comparison with dasatinib. In addition, the effect of Si306 and pro-Si306 was not affected by the presence of MDR phenotype. Both compounds did not change the P-gp expression at mRNA and protein level, differently from dasatinib. Indeed, after 72 h treatment, dasatinib increased P-gp expression. This implies that the application of our novel c-Src inhibitors could not further increase P-gp expression in MDR GBM cells. Moreover, Si306 and pro-Si306 induced significant suppression of P-gp activity and their effect was dose-dependent. They directly interacted with P-gp, showing significant P-gp inhibition after 30 min of treatment.

Other authors reported transient decrease of P-gp expression achieved by 1 μM dasatinib after 24 h in MCF-7 (MCF-7/Adr) cell line, reversing its MDR to doxorubicin after 72 h. This effect on P-gp expression enabled reversal of doxorubicin resistance by 3.35-fold when 0.5 μM dasatinib was applied for 72 h. Dasatinib inhibited the cell growth of (MCF-7/Adr) with low efficacy, reaching IC_50_ at 40 μM [60]. In our experimental setting of 72 h simultaneous combined treatment, 0.5 μM of Si306 and pro-Si306 reverted PTX resistance in MDR GBM cells by 3.34 and 7.98-fold, respectively. The sensitization of U87-TxR cells was achieved by concentrations that were significantly lower than those necessary for the optimal cell growth inhibition or P-gp inhibition induced by Si306 and pro-Si306. Therefore, even sub-cytotoxic concentrations of new c-Src inhibitors were able to increase PTX intracellular accumulation by inhibiting P-gp. It is possible that the inhibitory effect of drug and prodrug on P-gp activity increases over time enabling the enhancement of PTX efficacy. Stronger effects regarding P-gp inhibition and reversal of PTX resistance were observed after pro-Si306 application, indicating a more potent activity of prodrug against P-gp.

Human cytochrome P450 is a family of enzymes responsible for the essential biotransformation of xenobiotics [61]. Both P-gp and cytochrome P450 are involved in the first line of xenobiotic defense system, sometimes referred to as a cellular ADME system [62]. In addition, drug metabolism via the cytochrome P450 system has emerged as an important determinant in the occurrence of several drug interactions that can result in drug toxicity, reduced pharmacological effect, and adverse drug reactions. Predominant isoforms of cytochrome P450 involved in drug metabolism are CYP2C9, CYP2D6 and CYP3A4 which metabolize up to 80–90% of all xenobiotics [63,64,65,66]. In particular, it has been estimated that CYP3A4 metabolizes up to 50% of all reported drugs and a few of them are also metabolized by other isoforms. In the liver and small intestine, p-glycoprotein and CYP3A4 are both expressed and create a functional drug efflux-metabolism “alliance”, which establishes an absorption barrier against xenobiotics [67]. Moreover, it was shown that the second generation of P-gp inhibitors are metabolized by CYP3A4, thus competing with anticancer drugs for CYP-mediated clearing, finally leading to the adverse toxicity [62,68]. Coordinately, it is important to elucidate whether P-gp inhibitors (Si306 and its prodrug) may interact with cytochrome P450. Testosterone hydroxylation is in general used today as one of the characteristic assays for testing the inhibition of P450 isoform 3A4. The major product of testosterone metabolism is 6β-hydroxytestosterone but several other hydroxylations occur, including those at the 2β and 15β positions [69,70,71]. The evidence that the testosterone 6β-hydroxylation is not also inhibited by the two compounds at the highest concentration used clearly indicates that both Si306 and its prodrug did not interact with the CYP3A4.

It is well-known that drug permeability through biological membranes depends on factors such as plasma protein-binding, hydrophobicity and molecular size of the drug. Among these factors, protein-binding of the drug, which is closely-related to hydrophobicity, plays an important role in drug pharmacokinetics and pharmacodynamics. HSA is the major protein component of blood plasma (about 60%) and binds a number of relatively insoluble endogenous compounds. AGP primarily binds basic drugs (e.g., amines) and it is also reported to bind hydrophobic compounds [72]. For these reasons, the binding to albumin and alpha 1-acid glycoprotein of Si306 and pro-Si306 was evaluated. Both pro-Si306 and its parental drug Si306 showed high affinity for HSA and AGP plasmatic proteins (drugs that are highly bound are characterized by a K_D_ value less than 100 µM). For AGP, K_D_ values for Si306 and pro-Si306 were comparable, both being weak basic compounds. On the other hand, the very poorly water soluble [50] Si306 displayed a very high affinity for albumin, 60-fold greater than pro-Si306. This feature may suggest a plasmatic large fraction of bound drug, which could be the reason for its slow plasma clearance kinetics.

From our previous study, pro-Si306 emerged as an effective potential drug against GBM, but unfortunately is not suitable for an oral administration due to the complete hydrolysis into the GI apparatus (mainly stomach) [50]. On the other hand, Si306 can be administrated p.o. and this is one of the most important advantages in the use of TKIs, offering logistic flexibility and a good compliance in patients [73].

To prove that Si306 could be considered a good drug candidate for the treatment of GBM that is worthy of further development, a PK study after p.o. administration and i.v. injection has been performed.

The peak plasma concentration of Si306 was observed at 5 min for i.v. and 1.5 h for p.o. The plasma half-life (t_1/2_) resulted comparable between the two routes of administration and the oral bioavailability of Si306 has been estimated around 33.3% in mice, being higher than that reported for dasatinib (14% in mice) [74]. After both i.v. and p.o. administration Si306 was able to reach the brain and pass the BBB. In particular, after i.v. injection the maximum concentration into the brain (2.94 μg/g, Table 6) was observed after 5 min, while after oral administration a large amount of our drug has been detected (10.65 μg/g, Table 6) into the brain after 30 min. In both cases, the concentration of the compound slowly decreased during the next 24 h. Overall, our new results demonstrated a good absorption of Si306 by GI and, most interestingly, an optimal brain penetration, with a 12-fold higher AUC brain/plasma when Si306 was orally administered with respect to the i.v. injection. The higher and very fast brain accumulation of oral Si306 could be related to the greater inhibition of p-gp, and possibly saturation of p-gp sites at the BBB, which could be simply a consequence of the orally administrated dose, which was twice that of the i.v. [75].

In this context, being the Si306 considered able to block intestinal p-gp and since direct inhibition of p-gp in the small intestine was found to improve the oral bioavailability of p-gp substrates (dramatic increase in intestinal absorption of paclitaxel was observed when p-gp inhibitors were co-administrated [75]), the idea of combination with other drugs substrates of p-gp could be proposed.

Finally, in order to verify the safety of Si306 in vivo, an intravenously single-dose toxicity study was performed. The compound was very well tolerated by mice, even at a high dose (100 mg/Kg). Moreover, no mortality or changes in animal behavior were observed following the administration. At fourth day from the injection of Si306, mice were sacrificed, and their organs were analyzed. Any microscopic difference in the architecture of the main metabolic organs was observed, indicating very good tolerability and a lack of acute toxicity.

## 4. Materials and Methods

### 4.1. Drugs

A pyrazolo[3,4-*d*]pyrimidine derivative Si306 as well as its prodrug, pro-Si306, were previously synthesized and characterized [50]. Dasatinib as well as PTX and Dex-VER were purchased from Sigma-Aldrich (Milan, Italy and Taufkirchen, Germany suppliers). Dr. Sven Rottenberg from The Netherlands Cancer Institute in Amsterdam kindly provided TQ for our research purposes. Aliquots of 20 mM of both Si306 and pro-Si306 were dissolved in dimethyl sulfoxide (DMSO) and kept at room temperature. TQ was also dissolved in DMSO (10 µM stocks) but kept at −20 °C. PTX aliquots were stored at −20 °C in absolute ethanol (1 mM), while sterile water dilution of Dex-VER was kept at room temperature. Immediately before treatments, the aforementioned drugs were diluted in sterile water.

### 4.2. Cells and Culture Conditions

Human GBM cell lines U87 and LN-229 were purchased from the American Type Culture Collection, Rockville, MD, USA. U87-TxR cells were selected from U87 cells by continuous exposure to increasing concentrations of PTX [76]. LN-299, U87 and U87-TxR cells were grown in Minimum Essential Medium with 10% fetal bovine serum, 2 mM L-glutamine and 5000 U/mL penicillin, 5 mg/mL streptomycin solution. Cell lines were maintained at 37 °C in a humidified 5% CO_2_ atmosphere. They were sub-cultured at 72 h intervals using 0.25% trypsin/EDTA and 16,000 cells/cm^2^ were seeded into a fresh medium.

### 4.3. MTT Assay

MTT assay was used to assess the viability of U87 and U87-TxR cells (AppliChem GmbH, Taufkirchen, Germany). After reaching the confluence in 25 cm^2^ tissue flasks, U87 and U87-TxR cells were trypsinized. Cells were seeded at the optimal density determined in initial experiments: 4000 cell/well into flat-bottomed 96-well tissue culture plates. Following the overnight incubation, cells were treated with Si306, pro-Si306 and dasatinib (1–25 µM) for 72 h. In addition, the combined effects of Si306 and pro-Si306 with PTX were evaluated in MDR GBM cells. To that end, two concentrations of Si306 and pro-Si306 (0.2 μM and 0.5 μM) were combined with increasing concentrations of PTX (0.1–2 μM) in simultaneous treatment. The sensitization of U87-TxR cells to PTX was studied after 72 h. Briefly, MTT was added to each well in a final concentration of 0.2 mg/mL for 4 h to induce the formation of the formazan in cells with intact/viable mitochondria. Then, produced formazan was dissolved in DMSO, and the absorbance was measured at 540 nm on a microplate reader (LKB 5060-006 Micro Plate Reader, Vienna, Austria). IC_50_ values were calculated by nonlinear regression analysis using GraphPad Prism 6.0 for Windows (GraphPad Software, La Jolla, CA, USA).

### 4.4. RNA Extraction and Reverse Transcription (RT) Reaction

U87-TxR cells, untreated and treated with 5 μM of each compound Si306, pro-Si306 and dasatinib for 72 h were used to isolate total RNA and perform the RT reaction. Isolation was done by using Trizol^®^ reagent (Invitrogen Life Technologies, Waltham, MA, USA). Quantification of RNA and its quality determination were performed by spectrophotometry and by agarose gel electrophoresis, respectively. For the RT reactions, 2 µg of total RNA were used, while a high-capacity cDNA reverse transcription kit (Applied Biosystems, Waltham, MA, USA) was employed.

### 4.5. Quantitative Real-Time PCR

To assess mRNA *mdr1* expression level in U87-TxR cells upon 5 μM Si306, pro-Si306 and dasatinib treatments, the quantitative real time PCR (qRT-PCR) was used. The PCR reactions according to the manufacturer’s recommendations were performed in the presence of 100 ng cDNA combined with primers specific for *MDR1* and *ACTB*, as an internal control for normalization [77,78], and a Maxima SYBR Green/ROX qPCR Master Mix (Thermo Scientific, Waltham, MA, USA) on an ABI PRISM 7000 Sequence Detection System (Applied Biosystems, Waltham, MA, USA). Analyses of triplicate samples in terms of relative gene expression were completed using the 2^−ΔΔCt^ method [79].

### 4.6. Flow-Cytometric Analysis of P-Glycoprotein Expression

To assess the P-glycoprotein expression level in MDR GBM cells, flow-cytometry was employed. U87-TxR cells seeded in adherent 6-well plates were treated with 5 µM Si306, pro-Si306 or dasatinib for 72 h. Then, cells were collected by trypsinization, washed in PBS, and then directly immuno-stained by FITC-conjugated anti-P-gp antibody (BD Biosciences, Winnersh, Berkshire, UK). To discriminate the level of background fluorescence, an isotype control IgG2bκ (Abcam, Cambridge, United Kingdom) was used. The fluorescence of FITC-conjugated anti-P-gp was analyzed on assessed on the fluorescence channel 1 (FL1) of the CyFlow Space Partec flow-cytometer (Sysmex Partec GmbH, Görlitz, Germany). After gating to exclude the cell debris and dead cells, a minimum of 10,000 cells per sample was assayed. Summit Dako Software was used for the analyses of results obtained by flow-cytometry.

### 4.7. Rho 123 Accumulation Assay

Rho 123 accumulation was analyzed by flow-cytometry utilizing the ability of Rho 123, which is a substrate for P-glycoprotein, to emit fluorescence. The intensity of the fluorescence is proportional to Rho 123 accumulation in the cell. Studies were carried out with Si306, pro-Si306, dasatinib, Dex-VER and TQ in U87-TxR cells. U87 cells were used as a positive control for Rho 123 accumulation. In simultaneous treatment that lasted 30 min, 5 μM Rho 123 was applied along with 5 μM of Si306, pro-Si306 and dasatinib. To obtain IC_50_ values for P-gp inhibition, 5 μM Rho 123 was simultaneously applied with the increasing concentrations of Si306, pro-Si306, Dex-VER (1, 2, 5, 10 and 20 μM) and TQ (1, 2, 5, 10 and 20 nM). Samples were incubated 30 min at 37 °C in 5% CO_2_. At the end of the accumulation period, the cells were pelleted by centrifugation, washed with PBS and placed in cold PBS. The samples were kept on ice in a dark setting until the analysis on CyFlow Space Partec flow-cytometer (Sysmex Partec GmbH, Germany). The orange fluorescence of Rho 123 was assessed on fluorescence channel 2 (FLH-2) at 585 nM. A minimum of 10,000 events was assayed for each sample and the obtained results were analyzed using Summit Dako Software (ver. 4.3, Fort Collins, CO, USA).

### 4.8. Data Collection and Statistical Methods

Statistical analyses for P-gp inhibition and combination treatments were performed by two-way ANOVA test (GraphPad Prism 6 software) using Dunnett’s multiple comparisons test. Statistical significance was accepted if *p* < 0.05.

### 4.9. Binding Fluorimetric Assay

In order to determine the binding affinity for Si306 and pro-Si306 to HSA (human serum albumin) and AGP (alpha-1-acid glycoprotein), the protein fluorescence has been monitored by spectroscopy. The quantitative analysis was performed using black 96 multiwell plates: in each well, a fixed concentration of HSA or AGP (10 µM in phosphate buffer 1 mM) was added with different amounts of the tested compound (0.1 µM to 500 µM). Plates were gently shacked and after 30 min at room temperature for equilibration, spectra were recorded from 300 to 400 nm, after excitation at 295 nm, by using a Perkin Elmer EnVision Multilabel Reader 2014 spectrofluorimeter. Finally, spectra were acquired with EnVision Manager ver.1.13 software (Walthman, MA, USA). The obtained non fluorescence quenching percentages were plotted against drug concentrations and the relative K_D_ values were calculated using GraphPad software (version 6.0).

### 4.10. CYP450 Inhibition Study

The inhibition of cytochrome P450 3A4 isoform by Si306 and pro-Si306 was measured by determining its activity on the substrate testosterone.

The activity of CYP3A4 was evaluated by measuring the 6β-hydroxylated testosterone formation (CYP3A4-specific reaction). The amount of 6β-hydroxylated metabolite was assessed in the absence and presence of Si306, pro-Si306 and ketoconazole (testosterone concentration of 100 μM in phosphate buffer 0.025 M, pH 7.4). The compounds were solubilized in DMSO and added in a test tube to give final concentrations 1, 10, 25, 50 and 0.01, 0.05, 0.5, 5 μM, (DMSO did not exceed 2%). Ketoconazole was used as standard selective inhibitor of CYP3A4 isoenzyme [55]. Incubations were carried out in a mix containing human liver microsomes (1 mg/mL of protein) in phosphate buffer 0.025 M, pH 7.4, NADPH (200 μM) previously solubilized in a MgCl_2_ 48 mM solution. The final incubation volume was 0.25 mL. After a 40 min-incubation period at 37 °C, the reaction was stopped by adding 1 mL of acetonitrile in the presence of corticosterone 2 μM as internal standard. The samples were centrifuged for 20 min at 5000 rpm. Concentrations of testosterone and its metabolite 6β-hydroxytestosterone were assessed by the HPLC method with UV detection. Calibration curve for quantitative analysis of 6β-hydroxytestosterone in the samples was obtained using commercial 6β-hydroxytestosterone purchased from Sigma Aldrich. For the statistical analysis, GraphPad InStat 3.0 (GraphPad Software, San Diego, CA, USA) was used.

### 4.11. Animals

#### 4.11.1. For PK and BD Studies

Naive male BALB/C mice (weight 20 ± 2 g) were ordered from Charles River (Milan, Italy). All animals were pathogen free and approximately 4–6 weeks old when they arrived. The adaption period to the environment was not less than seven days. All the procedures used on animals in this study were approved by Institutional Animal Use and Care Committee at Università Cattolica del S. Cuore, Rome and Università degli Studi di Siena and authorized by the Italian Ministry of Health, according to Legislative Decree 116/92, which implemented the European Directive 86/609/EEC on laboratory animal protection in Italy. Methods for all the conducted experiments were performed in accordance with regulations, standards and guidelines of the Animal Use and Care Committee of Università Cattolica del S. Cuore, Rome and Università degli Studi di Siena.

#### 4.11.2. For Acute Toxicity Studies

Male BALB/c mice (20 ± 2) g, supplied by Harlan Italy S.r.l., San Pietro al Natisone, Udine, Italy, were acclimated at 25 °C and 55% of humidity under natural light/dark conditions for 1 week before the proposed experiments. Animal welfare was routinely checked by veterinarians of the Service for Animal Welfare and all efforts were made to minimize suffering.

### 4.12. In Vivo Administration of Si306

Si306 was dissolved in a mixture of Tween80 (10% v/v), benzyl alcohol (1% v/v) and a 10 mM solution of citric acid. The compound was administered intravenously as a single dose of 25 mg/kg in 200 μL of volume. At several time points (0.08, 0.25, 0.5, 1, 2, 4, 8, 24 h), after drug administration, mice were treated i.p. with heparin (5000 U/kg) and sacrificed under CO_2_. Five animals were used for each time point. Blood, brain, liver and kidneys were collected for the following quantitative analysis. Approximately 500–600 μL of blood was collected from each animal and transferred to a tube containing 10 μL of heparin and mixed briefly.

For the oral PK studies, mice received a single dose of 50 mg/kg of Si306 (dissolved in the same vehicle used for i.v. studies) in 400 μL of final volume by gavage. At nine time-points (0.25, 0.5, 1, 1.5, 2, 3, 4, 8, 24 h) after administration, mice were sacrificed under CO_2_. Blood and organs were collected and analyzed by LC-MS. The pharmacokinetic parameters, including area under concentration-time curve (AUC), maximum plasma concentration (C_max_), half-time (t_1/2_), apparent volume of distribution (V), plasma clearance (CL) and mean residence time (MRT), were calculated by non-compartmental analysis using PKSolver software (ver. 1.0, Nanjing, China) [80].

#### 4.12.1. Sample Preparation

Blood samples were centrifuged at 5000 rpm for 20 min to separate the plasma fraction, which was subsequently collected in a test tube. Acetonitrile (1 mL, with Si34 as internal standard at the concentration of 5 μg/mL) was added to each sample to denature proteins. Samples were centrifuged at 5000 rpm for 20 min, and the supernatant was recovered, dried under vacuum, solubilized again in methanol (100 μL), and analyzed. Brain and the other organs were homogenized using a T10 basic ULTRA-TURRAX^®^ homogenizer (Bioclass, Pistoia, Italy); in order to extract the compound from the tissue, acetonitrile internal standard solution was added, and the homogenate was centrifuged at 5000 rpm for 20 min. The supernatant was recovered, filtered and analyzed by LC-MS. The quantification of each compound was performed by reference to the appropriate calibration curve. Representative LC-MS chromatograms for Si306 and for Si34 are reported in Figure S2 and Figure S3 respectively. Mass spectra are also shown in Figure S4.

#### 4.12.2. Recovery and Matrix Effect

The matrix effect was evaluated in samples by comparing the peak areas of the Si306 reference solutions reconstituted in blank organ extracts (for plasma, brain, liver and kidneys) against those of the analyte dissolved in mobile phase. The recovery of Si306 was evaluated by comparing the peak area of Si306 in spiked plasma and other organs samples with the peak areas of analyte spiked after extraction into plasma or organs’ extracts [81]. The matrix effect of the IS was evaluated in a similar manner.

#### 4.12.3. UV/HPLC-MS Instrumentation and Analysis Condition

LC analyses were performed by using Agilent 1100 LC/MSD VL system (G1946C) (Agilent Technologies, Palo Alto, CA, USA) constituted by a vacuum solvent degassing unit, a binary high-pressure gradient pump, a 1100 series UV detector, and a 1100 MSD model VL benchtop mass spectrometer. MSD single-quadrupole instrument was equipped with the orthogonal spray API-ES (Agilent Technologies, Palo Alto, CA, USA). The pressure of the nebulizing gas and the flow of the drying gas (nitrogen used for both) were set at 40 psi, 9 L/min, respectively. The capillary voltage, the fragmentor voltage, and the vaporization temperature were 3000 V, 70 V, and 350 °C, respectively. MSD was used in the positive ion mode. Spectra were acquired over the scan range m/z 50–1500 using a step size of 0.1. Chromatographic analysis for CYP3A4 interaction studies was performed using a Phenomenex Luna C18 100A column (250 × 4.6 mm, 5 μm particle size) at room temperature, at flow rate of 0.6 mL/min, and injection volume was 20 μL, operating with a gradient elution of acetonitrile (ACN) and water (H_2_O): t = 0 min ACN 0%, t = 2 min ACN 0%, t = 40 min ACN 70%, t = 45 min ACN 70%, t = 50 min ACN 0%. UV detection was monitored at 254 nm. Chromatographic analysis for Si306 and its internal standard Si34 was performed using a Kinetex EVO C18 100A column (150 × 4.6 mm, 5 μm particle size) at room temperature, at flow rate of 0.6 mL/min, and injection volume was 20 μL, operating with a gradient elution of acetonitrile (ACN) and water (H_2_O): t = 0 min ACN 0%, t = 3 min ACN 0%, t = 12 min ACN 98%, t = 18 min ACN 98%. Representative LC-MS chromatograms and mass spectra have been reported in Supporting Information.

### 4.13. Evaluation of Acute Safety Profile in Mice

The study design includes three groups of five mice each. All mice were injected with compound through the tail vein in a single systemic dose (0.05 mL per dose). The test compound was solubilized in PBS/25% DMSO. The first group was treated with dose levels of 50 mg/kg; the second received a high-dose level of 100 mg/kg, whereas the third was inoculated with the only vehicle as the control. All mice were monitored for survival and the presence of any drug-related adverse effect (local signs of inflammation, weight loss, diarrhea, and behavioral alterations) throughout the experimental period. Mice were sacrificed 4 days after treatment, and liver, kidney, and brain tissue specimens were aseptically removed, fixed in 4% formaldehyde solution, and processed for paraffin embedding. Histopathological changes in tissue specimens were evaluated in 10 randomly selected tissue sections from each organ for all the samples. Sections cut at 2 μm were stained with hematoxylin and eosin (H&E) and analyzed by a pathologist with an optical microscope (Leica, Wetzlar, Germany).

## 5. Conclusions

As a continuation of efforts in the development and optimization of new TKIs active against GBM, in the present study we described the further in vitro and in vivo characterization of Src inhibitor pyrazolo[3,4-*d*]pyrimidine derivatives. The identification of TKIs whose activity could not be affected by developed multidrug resistance mechanisms is an urgent need. The efficacy of many TKIs is diminished by the fact that they are P-glycoprotein substrates. Herein, we showed that the sensitivity of multidrug resistant GBM cells was not compromised by pyrazolo[3,4-*d*]pyrimidine derivatives treatment. Moreover, we reported that Src inhibitors, Si306 and its prodrug possess valuable characteristics for GBM treatment. Both compounds are able to suppress P-glycoprotein activity without inducing its expression in multidrug resistant GBM cells. In addition, Si306 and pro-Si306 do not interact with CYP3A4 and possess the ability of binding to plasma proteins. The penetration of Si306 in mouse brain after oral administration was confirmed to be optimal, while toxicology studies showed a good tolerability profile of this agent. All these features imply that the Src inhibitor Si306 could be considered as a valuable strategy for GBM treatment alone or in combination with other chemotherapeutics. Furthermore, Si306 could facilitate the accumulation of co-administered drugs into the brain and GBM site, due to its high activity against P-gp present in BBB.

## Figures and Tables

**Table 1:** Sensitivity of glioblastoma multiforme (GBM) cells to c-Src inhibitors expressed as half maximal inhibitory concentration (IC_50_) values ± S.D. (µM), calculated from three independent experiments (*n* = 3).

Compounds	U87	U87-TxR	LN-229
Dasatinib	6.143 ± 0.464	8.516 ± 0.691	24.682 ± 1.027
Si306	3.081 ± 0.260	4.775 ± 0.322	8.008 ± 0.574
Pro-Si306	3.045 ± 0.343	3.419 ± 0.359	3.074 ± 0.272

**Table 2:** P-gp inhibition calculated by Rho 123 accumulation in cell.

P-gp Inhibition	TQ ^a^	Si306	Pro-Si306	Dex-VER
**IC_50_ (µM)**	0.343 ± 0.017	8.496 ± 0.161	3.748 ± 0.060	0.735 ± 0.029

IC_50_ ± S.D. (*n* = 3); ^a^ IC_50_ ± S.D. values for tariquidar (TQ) are expressed in nM

**Table 3:** Relative reversal of PTX resistance in U87-TxR cells induced by Si306 and pro-Si306.

Compounds	IC_50_ ± S.D. for PXT (µM)	Relative Reversal
	2.840 ± 0.358	
Si306		
0.2 µM	2.449 ± 0.121	1.16 ^ns^
0.5 µM	0.850 ± 0.088	3.34 **
Pro-Si306		
0.2 µM	0.635 ± 0.103	4.47 ***
0.5 µM	0.356 ± 0.072	7.98 ***

Ns, non-significant; **, *p* ≤ 0.01; ***, *p* ≤ 0.001

**Table 4:** Plasmatic proteins binding parameters for Si306 and pro-Si306.

Compounds	Parameters	Si306	Pro-Si306
HSA	K_D_^a^ (µM)	0.35 ± 0.1	21.45 ± 4.5
	B_MAX_^b^	61.05 ± 2.8	78.00 ± 4.2
AGP	K_D_ (µM)	11.49 ± 1.0	9.28 ± 0.7
	B_MAX_	88.38 ± 1.8	96.69 ± 1.5

^a^ Ligand concentration that binds to half the receptor sites at equilibrium; ^b^ maximum number of binding sites.

**Table 5:** Matrix effect and recovery of Si306 in mice plasma, brain, liver and kidneys (*n* = 3).

µg/mL	Matrix Effect (%)				Recovery (%)			
	Plasma	Brain	Liver	Kidneys	Plasma	Brain	Liver	Kidneys
0.1	150	-	-	-	102.6	-	-	-
1	139.3	137.9	118.8	99.0	84.7	76.7	80.7	89.7
10	140.2	-	-	-	73.4	-	-	-
50	142.1	87.9	112.9	92.2	70.5	111.6	70.2	86.0
100	-	88.2	106.3	101.2	-	123.1	85.3	80.8

**Table 6:** Plasma and brain pharmacokinetic parameters for Si306 ^a.^

Parameters	Unit	PLASMA		BRAIN	
Route		i.v.	p.o.	i.v.	p.o.
Dose	mg/kg	25	50	25	50
C_max_^b^	µg/mL	25.45	2.97	2.94	10.65
T_max_^c^	h	0.08	1.5	0.08	0.5
MRT ^d^_0__→__∞_	h	3.78	5.70	1.48	1.20
AUC ^e^_0→__∞_	µg/mL × h	22.75	15.17	2.15	17.97
AUC ^e^_0→24 h_	µg/mL × h	22.30	14.48	2.14	17.96
V ^f^_z/F_	L/Kg	8.17	28.32	44.04	8.44
CL ^g^_z/F_	L/h/Kg	1.10	3.29	11.60	2.78
t_1/2_^h^	h	5.15	5.96	2.63	2.10

^a^ Calculated with PKSolver; ^b^ C_max_: maximum concentration observed. ^c^ T_max_: time of maximum concentration observed. ^d^ MRT: mean residence time. ^e^ AUC: area under the curve. ^f^ V: volume of distribution. ^g^ CL: clearance. ^h^ t_1/2_: half-life. Pharmacokinetic data were evaluated using a non-compartment model.

## Supplementary Materials

The following are available online at https://www.mdpi.com/2072-6694/11/6/848/s1, Figure S1: Rho 123 accumulation in U87-TxR cells treated with increasing concentrations of TQ (A), Dex-VER (B), Si306 (C) and pro-Si306 (D), Figure S2: LC-MS chromatogram for Si306, Figure S3: LC-MS chromatogram for Si34, used as internal standard for Si306, Figure S4: Mass spectra of (a) Si306 and (b) Si34. Table S1: Sensitivity of glioblastoma multiforme (GBM) cells to Si306 and pro-Si306 expressed as IC_50_ values ± S.D.
