# Supplementary material for: A New Strategy for Glioblastoma Treatment: In Vitro and In Vivo Preclinical Characterization of Si306, a Pyrazolo[3,4-d]Pyrimidine Dual Src/P-Glycoprotein Inhibitor
Source: Cancers (Basel). 2019 Jun 19;11(6):848. doi: 10.3390/cancers11060848 (PMC6628362; doi:10.3390/cancers11060848)
Supplement: Supplementary file 1 [file cancers-11-00848-s001.pdf]

Supplementary Materials: A New Strategy for Glioblastoma Treatment: In Vitro and In Vivo Preclinical Characterization of Si306, a Pyrazolo[3,4-*d*]Pyrimidine Dual Src/P-Glycoprotein Inhibitor

Anna Lucia Fallacara, Claudio Zamperini, Ana Podolski-Renić, Jelena Dinić, Tijana Stanković, Marija Stepanović, Arianna Mancini, Enrico Rango, Giulia Iovenitti, Alessio Molinari, Francesca Bugli, Maurizio Sanguinetti, Riccardo Torelli, Maurizio Martini, Laura Maccari, Massimo Valoti, Elena Dreassi, Maurizio Botta, Milica Pešić and Silvia Schenone

1. Cell Growth Inhibition by Dasatinib, Si306 and Pro-Si306

GB cell lines BT138, T98G and A172 were treated with Si306 and pro-Si306 at concentrations ranging from 1 to 100 μM, or with DMSO as a control. Cell viability was evaluated 72 h after treatment for T98G and A172 cell lines and after 24 h for BT-138. In table S1 the IC_{50} values are reported.

Table S1. Sensitivity of glioblastoma multiforme (GBM) cells to Si306 and pro-Si306 expressed as IC_{50} values ± S.D.

Compounds	BT-138 μM (24 h)	T98G μM (72 h)	A172 μM (72 h)
Si306	72.58 ± 6.21	0.46 ± 0.02	1.69 ± 0.60
Pro-Si306	30.71 ± 4.75	/	/

2. Rho 123 Accumulation (Proportional to the Level of P-gp Inhibition)

The inhibition of P-gp function illustrated by dose-dependent accumulation of Rho 123. Rho 123 accumulation was assessed in untreated non-MDR (U87) and MDR (U87-TxR) cells as well as in U87-TxR cells treated with increasing concentrations of Si306 (C), pro-Si306 (D), Dex-VER (B; 1, 2, 5, 10, 20 μM) and TQ (A; 1, 2, 5, 10, 20 nM). This Supplementary Figure S1 shows the methodology used for the calculation of IC50 values for P-gp inhibition presented in Figure S3 and Table S2.

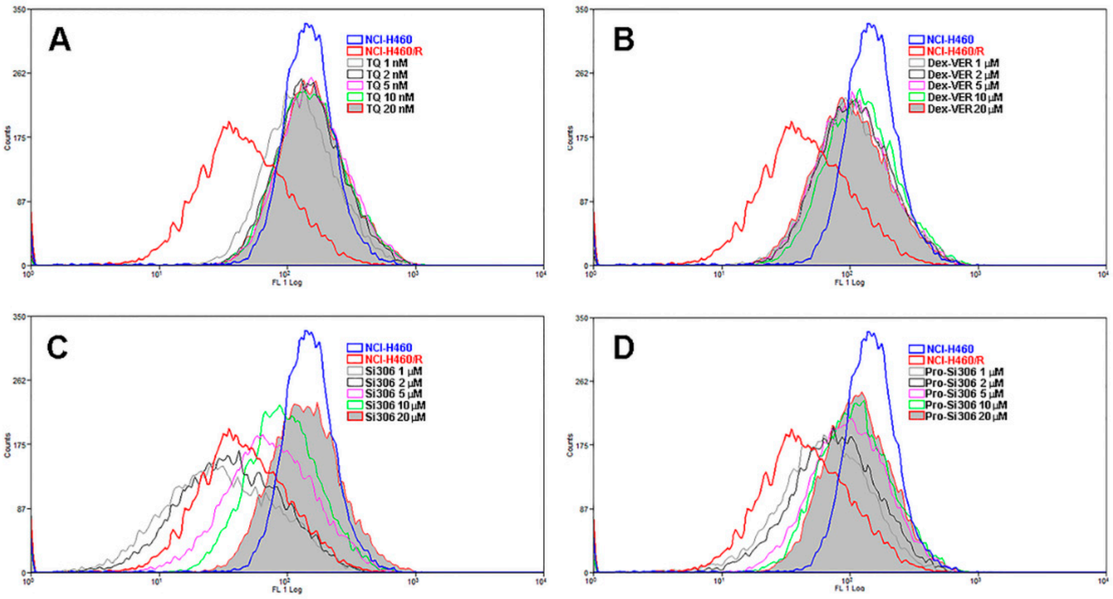

Figure S1. Rho 123 accumulation in U87-TxR cells treated with increasing concentrations of TQ (**A**), Dex-VER (**B**), Si306 (**C**) and pro-Si306 (**D**).

2. Si306 HPLC/MS Analysis

Si306 has been analyzed by HPLC-MS, using Si34 as internal standard. Representative LC-MS chromatograms (single ion monitoring) for Si306 and for Si34 are reported in Figure S3 and S4 respectively. In addition, mass spectra are shown in Figure S4.

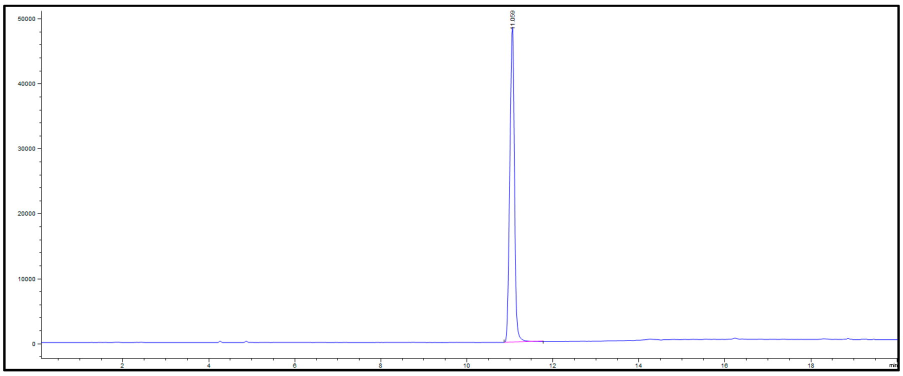

Figure S2. LC-MS chromatogram for Si306**.**

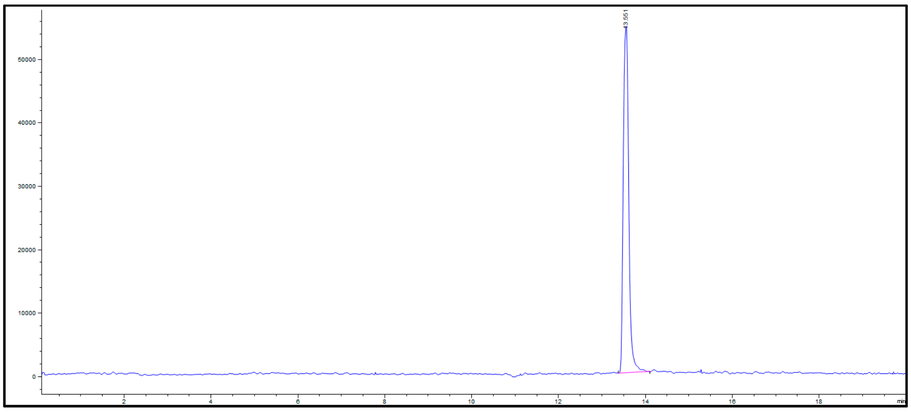

Figure S3. LC-MS chromatogram for Si34, used as internal standard for Si306.

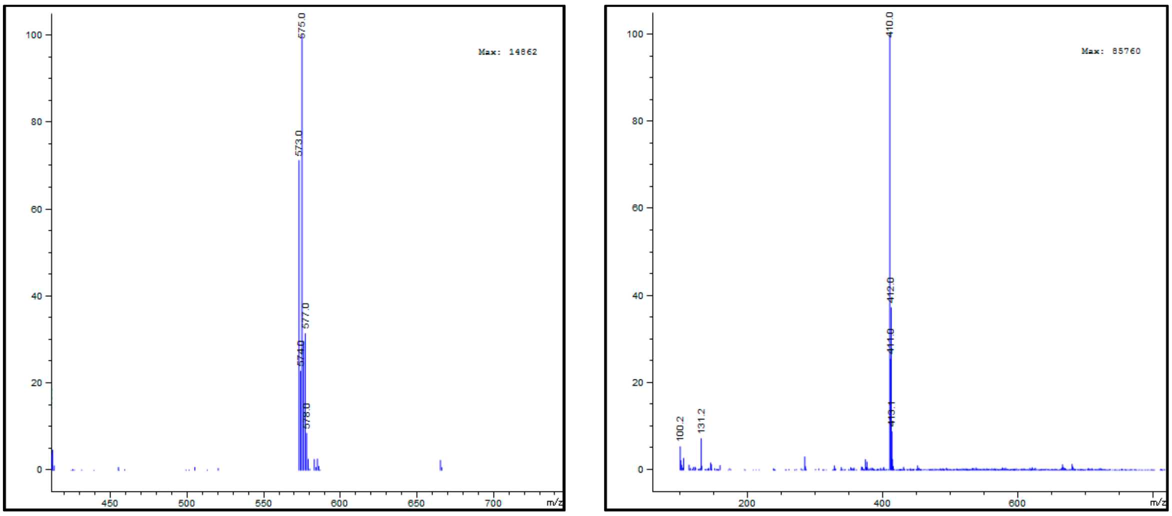

(a) **(b)**

Figure S4. Mass spectra of (**a**) Si306 and (**b**) Si34.
